# Extracellular matrix production and oxygen diffusion regulate chemotherapeutic response in osteosarcoma spheroids

**DOI:** 10.1002/cam4.70239

**Published:** 2024-09-20

**Authors:** Isabel S. Sagheb, Thomas P. Coonan, R. Lor Randall, Katherine H. Griffin, J. Kent Leach

**Affiliations:** ^1^ Department of Biomedical Engineering University of California Davis California USA; ^2^ Department of Orthopaedic Surgery UC Davis Health Sacramento California USA; ^3^ School of Veterinary Medicine University of California Davis California USA

**Keywords:** chemoresistance, chemotherapy, hypoxia, osteosarcoma, tumorigenesis

## Abstract

**Background:**

Osteosarcoma (OS) survival rates and outcome have not improved in 50 years since the advent of modern chemotherapeutics. Thus, there is a critical need for an improved understanding of the tumor microenvironment to identify better therapies. Extracellular matrix (ECM) deposition and hypoxia are known to abrogate the efficacy of various chemical and cell‐based therapeutics. Here, we aim to mechanistically investigate the combinatorial effects of hypoxia and matrix deposition with the use of OS spheroids.

**Methods:**

We use two murine OS cell lines with differential metastatic potential to form spheroids. We form spheroids of two sizes, use ascorbate‐2‐phosphate supplementation to enhance ECM deposition, and study cell response under standard (21% O_2_) and physiologic (5% O_2_) oxygen tensions. Finally, we examine chemotherapeutic responses to doxorubicin treatment.

**Results:**

ECM production and oxygen tension are key determinants of spheroid size through cell organization based on nutrient and oxygen distribution. Interestingly, highly metastatic OS is more susceptible to chemotherapeutics compared to less metastatic OS when matrix production increases. Together, these data suggest that dynamic interactions between ECM production and oxygen diffusion may result in distinct chemotherapeutic responses despite inherent tumor aggressiveness.

**Conclusion:**

This work establishes OS spheroids as a valuable tool for early OS tumor formation investigation and holds potential for novel therapeutic target and prognostic indicator discovery.

## Graphical abstract

I describe an in vitro investigation to study the influence of extracellular matrix (ECM) content and spheroid diameter (as a surrogate for oxygen tension) on the efficacy of chemotherapy in osteosarcoma (OS). Interestingly, highly metastatic OS was more susceptible to chemotherapeutics compared to less metastatic OS when matrix production increased. These data suggest that dynamic interactions between ECM production and oxygen diffusion may result in distinct chemotherapeutic responses despite inherent tumor aggressiveness.

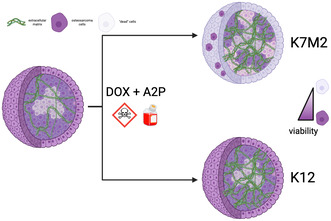

## 1 INTRODUCTION

Osteosarcoma (OS) is the most common bone cancer in children and adolescents, resulting in the formation of malignant osteoid.^1^, ^2^ These tumors are highly aggressive with very high metastatic potential. Doxorubicin‐based chemotherapy remains the backbone of systemic treatment with no improvement in survival over the past 50 years.^3^ This disappointing absence of clinical progress mandates further mechanistic investigation to improve our understanding of primary tumor development and the events leading to metastasis.

The majority of existing research on OS is conducted under standard culture conditions (21% O_2_) and on tissue culture plastic (TCP),^4^, ^5^ which fails to mimic the native environment of OS. As a sarcoma, OS is characterized by bulk stromal tissue formation that arises in the bone marrow niche and features an oxygen tension of 5% O_2_.^6^ Furthermore, hallmarks of aggressive OS involve selective matrix‐related changes that are clinically recognized as rapid tumor growth through matrix deposition that outpaces the nutrient and vascular supply, resulting in necrotic, hypoxic lesions.^1^ This, in turn, can reduce drug diffusion and targeting towards those regions.^1^, ^2^ Indeed, this close relationship between endogenous, interstitial matrix deposition and oxygen tension is widely studied in many solid tumors including OS. Many molecular and genetic studies show that master regulatory signaling pathways such as NOTCH and WNT,^7^ which are known under homeostasis to regulate not only matrix cues but also proliferation and differentiation, are also disrupted by hypoxic conditions, largely through the HIF signaling pathway.^8^ Indeed, multiple components of these pathways, from direct ligand targeting to microRNA biogenesis, are under investigation as potential therapeutics.^9^, ^10^ However, the vast majority of these studies have not modeled the hypoxic, three‐dimensional (3D) tumor microenvironment adequately, resulting in inconsequential conclusions and no appreciable change in clinical outcomes.

In the last 20 years, techniques to better model cancer environments have become a focus of the research field.^5^ Going beyond genetic interrogations, these techniques largely aim to characterize the role of various extracellular matrix (ECM) cues, such as chemical and mechanical signals, through 3D culture with engineered scaffolds.^5^, ^11^ Though limited applications exist for OS, multiple groups report significant changes to both primary tumor and immortalized OS cell line behavior under 3D conditions.^11^, ^12^ For example, OS cultured on hydroxyapatite scaffolds,^13^ in an engineered bone marrow environment,^14^ and collagen hydrogels containing cancellous bone, laminin, and fibronectin,^15^ have all shown stark differences in tumorigenic behavior compared to cells cultured in monolayer on TCP. Cellular aggregates formed from human OS cell lines were used to investigate responses to various clinically relevant chemotherapeutic regimes, yet these studies did not interrogate responses in conjunction with oxygen tension and matrix production.^16^ Mechanistic studies also show that stiffness‐driven changes that arise with ECM remodeling alter OS proliferation, differentiation, and immune regulation.^17^, ^18^ Thus, the study of OS in 3D is necessary to accurately evaluate OS tumor formation and development, yet there are few reports that seek to combine this approach while also interrogating oxygen‐related changes.

Here, we examine the cell‐ECM interactions of primary OS tumorigenesis under physiologically relevant oxygen tensions with the use of OS aggregates, known as spheroids. Using two murine OS cell lines with differential metastatic potential, we formed spheroids of two different sizes, studied them under standard (21% O_2_) and physiologic (5% O_2_) oxygen tensions, and examined chemotherapeutic responses to doxorubicin treatment. We also sought to understand the role of matrix deposition in OS tumorigenesis, and thus enriched endogenous matrix production in spheroids by media supplementation with ascorbate‐2‐phosphate (A2P). We hypothesize that spheroids will exhibit increases in pro‐tumorigenic markers, including proliferation and chemoresistance, as a function of metastatic potential and oxygen tension. Furthermore, we hypothesize that ECM deposition within these spheroids regulates these behaviors.

## 2 METHODS

### 2.1 Cell culture

Highly metastatic OS (K7M2, ATCC, Manassas, VA) and less metastatic OS (K12, courtesy of Dr. Kurt Weiss, University of Pittsburgh Medical Center) were expanded and maintained under standard (21% O_2_) culture conditions. Cells were cultured in DMEM (Invitrogen, Carlsbad, CA) supplemented with 10% fetal bovine serum (FBS) (Atlanta Biologics, Flowery Branch, GA) and 1% penicillin–streptomycin (P/S) (Gemini Bio Products, West Sacramento, CA).

### 2.2 Spheroid formation and culture

OS cells were formed into spheroids composed of either 5,000 (5 K) cells or 10,000 (10 K) cells. Spheroids were formed using a previously described forced aggregation method.^19^, ^20^ Briefly, OS cells (either 1.45×10^5^ cells/mL for 5,000 cell spheroids or 2.9×10^5^ cells/mL for 10,000 cell spheroids) were pipetted into 1.5% agarose molds, each containing 29 microwells, in 24‐well plates, and the plates were centrifuged at 500 × *g* for 8 min. Plates were maintained statically in standard or physiologic culture conditions for 72 h to form spheroids, after which media was refreshed immediately following this incubation and every 2–3 days for the duration of the study. Spheroid formation after 72 h is defined as the “Day 0” timepoint. Due to their small size, unless otherwise stated, biochemical quantifications are presented where each well of 29 spheroids serves as a pooled replicate. For endogenous enrichment of matrix deposition, media was supplemented with 50 μg/mL ascorbate‐2‐phosphate (A2P, Sigma‐Aldrich, St. Louis, MO) during and after formation. For chemotherapeutic response studies, media was supplemented with 0.1 μM doxorubicin (Neta Scientific, Hainesport, NJ).

### 2.3 Cell proliferation and viability characterization

Metabolic activity was quantified with an alamarBlue assay (ThermoFisher Scientific, Waltham, MA) prior to DNA quantification. Samples were incubated with the alamarBlue reagent for 1.5 h, analyzed, washed twice with PBS, then collected in 300 μL passive lysis buffer (Promega Madison, WI). DNA content was determined using the Quant‐iT PicoGreen DNA Assay Kit (Invitrogen, Carlsbad, CA). Cell viability was evaluated by a Live/Dead assay with calceinAM and propidium iodide staining (Invitrogen). Samples were imaged with a confocal microscope (Leica Stellaris 5, Leica Microsystems, Deerfield, IL) and representative images were assessed with ImageJ.

### 2.4 Histology

Spheroids were fixed in 10% buffered formalin, embedded in HistoGel (ThermoFisher Scientific), then paraffin embedded and sectioned at 5 μm thickness. Sections were stained with hematoxylin and eosin (H&E) and imaged with 10X and 20X objectives using a Nikon Eclipse TE2000U microscope. To visualize regions with oxygen tensions lower than 1.3%^21^, ^22^ within the spheroids, pimonidazole hydrochloride (200 μM; Hypoxyprobe; Chemicon, Temecula, CA) was added to the spheroids and incubated under standard or physiologic culture conditions for 2 h.^23^ Spheroids were then fixed and processed as described above. Samples were rehydrated, exposed to heat‐mediated antigen retrieval with sodium citrate buffer for 20 min, and incubated in blocking buffer composed of 10% goat serum and 10 mg/mL Bovine Serum Albumin (BSA) at room temperature for 30 min. Primary detection for pimonidazole hydrochloride was performed with a 1:100 dilution of the conjugated rat monoclonal anti‐pimonidazole IgG included in the kit, and cells were counterstained with DAPI. Other slides were rehydrated and exposed to enzyme mediated antigen retrieval with Proteinase K at 37°C for 30 min. Samples were blocked as described before, after which they were incubated with recombinant anti‐integrin α5 antibody (ab112183, Abcam, Cambridge MA) at a concentration of 1:100 overnight at 4°C. Slides were then treated with a secondary goat anti‐rabbit antibody conjugated with AF488 (ab150081, Abcam) at a concentration of 1:200 for 1 h at room temperature, and again counterstained with DAPI.

### 2.5 Collagen quantification

Collagen content was evaluated with a hydroxyproline kit (Chrondrex, Woodinville, WA), where hydroxyproline comprises an estimated 14% of total collagen amino acid content.^24^ Samples were collected in 5 M HCl, hydrolyzed at 120°C for 26 h, and prepared per manufacturer instructions.

### 2.6 Mechanical characterization

Spheroid compressive storage moduli were examined using a MicroTester G2 (CellScale, Waterloo, ON).^25^ Individual spheroids of approximately 500–600 μm were loaded onto an anvil in a water bath filled with PBS. The spheroids were compressed by 25% of their diameter by a stainless‐steel platen attached to a tungsten microbeam over 30 s. Microbeams with 0.2032 diameter were used for all tests. Displacement and force were tracked via MicroTester software. The linear region of the compressive modulus versus nominal strain graph was recorded as the calculated modulus.

### 2.7 Statistics

Data are presented as mean ± standard deviation. Shapiro–Wilk normality tests and F‐tests to compare variances were performed, and statistical analysis was conducted with Prism 10.2.0 (GraphPad, San Diego, CA) software utilizing either unpaired t‐test assuming Gaussian distribution and one‐way or two‐way analysis of variance (ANOVA) with post hoc Tukey's test depending on the number of groups and comparisons. Groups with different letters indicate statistically significant differences (*p* < 0.05), while groups with the same letters are not significant; ns denotes no significance among groups.

## 3 RESULTS

### 3.1 Spheroids formed with K7M2 and K12 cells require extended formation time

Spheroid formation, regardless of technique, is concluded when self‐assembled cell aggregates have deposited adequate extracellular matrix (ECM) for mechanical stability without disintegration.^26^ For most adherent cell types, this process is complete after 48 h. However, spheroids formed with K12 cells were neither stable nor spherical even at 72 h (Figure S1). Surprisingly, we noted significant cell migration up the microwell wall beginning as early as 24 h after centrifugation. To encourage increased cell–cell interactions, we attempted spheroid formation with lower cell densities, higher agarose percentages, and increased centrifugation speeds (*data not shown*), none of which altered this behavior. Instead, we found that artifact from the 3D printed negative molds^19^ created “steps” within the microwells. These steps facilitated K12 migration and egress from the focal center where spheroids normally form. This was remedied with the use of molds that were formed by casting in agarose^20^ instead of 3D printing, thus eliminating the printed artifacts. However, it is important to note that this original migratory behavior is quite unusual and was an early indication that cell signaling and matrix deposition in K12s may contribute to cancerous processes. This was also validated when, even with the revised molds, we found that OS spheroids still required an extended formation time of 72 h before becoming mechanically stable without disintegration.

### 3.2 OS spheroids have similar diameters regardless of initial cell seeding density

**FIGURE 1:**
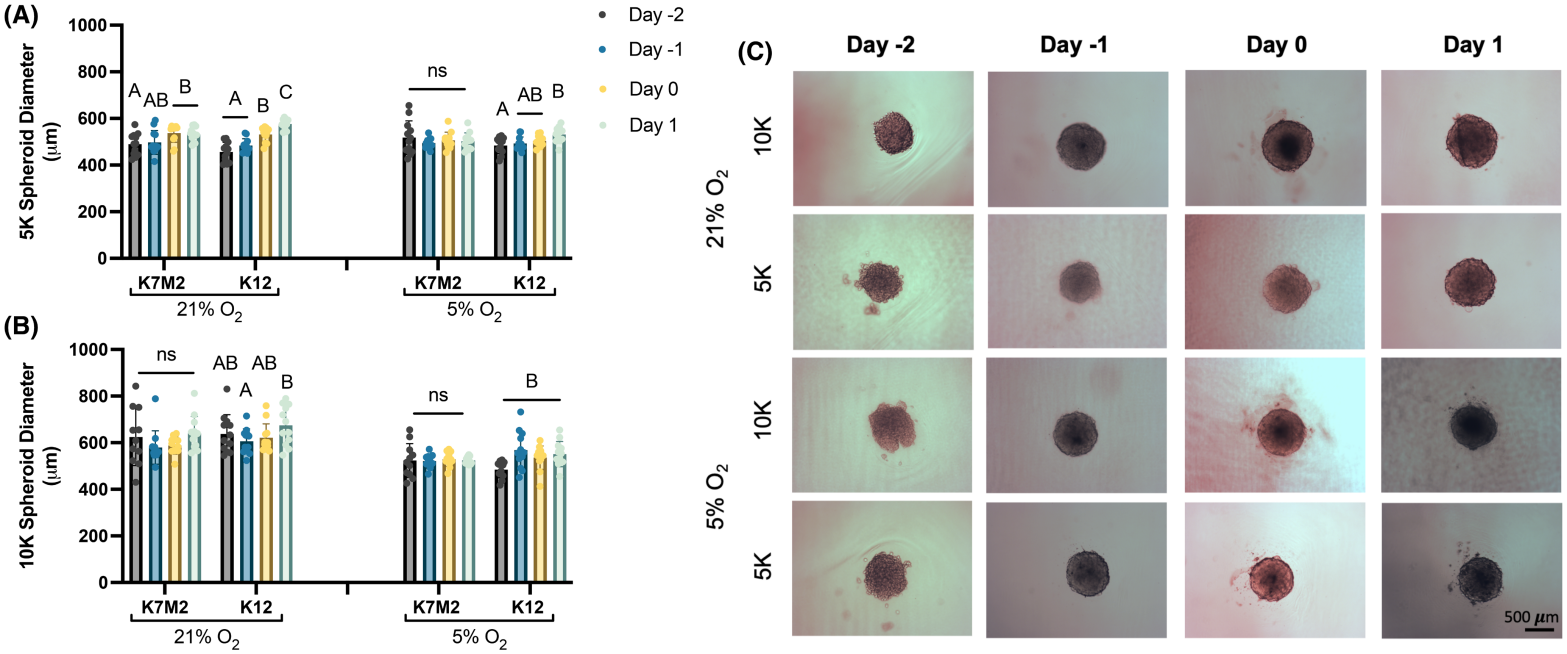
OS spheroids have similar diameters regardless of initial cell density. OS spheroid diameter during formation of spheroids formed with 5,000 (5 K) (A) or 10,000 (10 K) (B) cells per spheroid. (C) Representative brightfield microscopy images of spheroids during formation. Scale bar = 500 μm. Data are mean ± SD (*n* = 10–12 single spheroids, distributed over at least three separate wells). Groups with statistically significant differences based on two‐way ANOVA do not share the same letters; ns denotes no significance among groups.

We evaluated OS cell behavior during spheroid formation. To investigate how cell density and oxygen tension affected early OS spheroid formation, we formed spheroids of two densities, 5,000 cells/spheroid (5 K) and 10,000 cells/spheroid (10 K) and under two oxygen tensions, 21% and 5% O_2_. We measured the diameter of all spheroids (Figure 1A,B) from brightfield images (Figure 1C). Unlike other cells of mesenchymal origin,^26^ OS cells did not compact throughout the course of spheroid formation. Rather, spheroids formed with K7M2 cells generally did not change size over time, and spheroids formed with K12 cells slightly increased in diameter. Surprisingly, we found no significant differences between spheroids made with 5,000 and 10,000 cells, suggesting an autonomous rearrangement to optimize cell contacts and nutrient distribution. This is further supported by the significant difference we detected in spheroid diameter as a function of oxygen tension (Figure S2) where spheroids, regardless of cell type and density, show a 10% increase in size when formed under 21% O_2_ compared to 5% O_2_ (*p* = 0.0069). Together, these data suggest that spheroids formed under 21% O_2_ are larger due to increased oxygen availability and subsequent diffusion.

### 3.3 Spheroids with 10,000 OS cells have decreased viability

**FIGURE 2:**
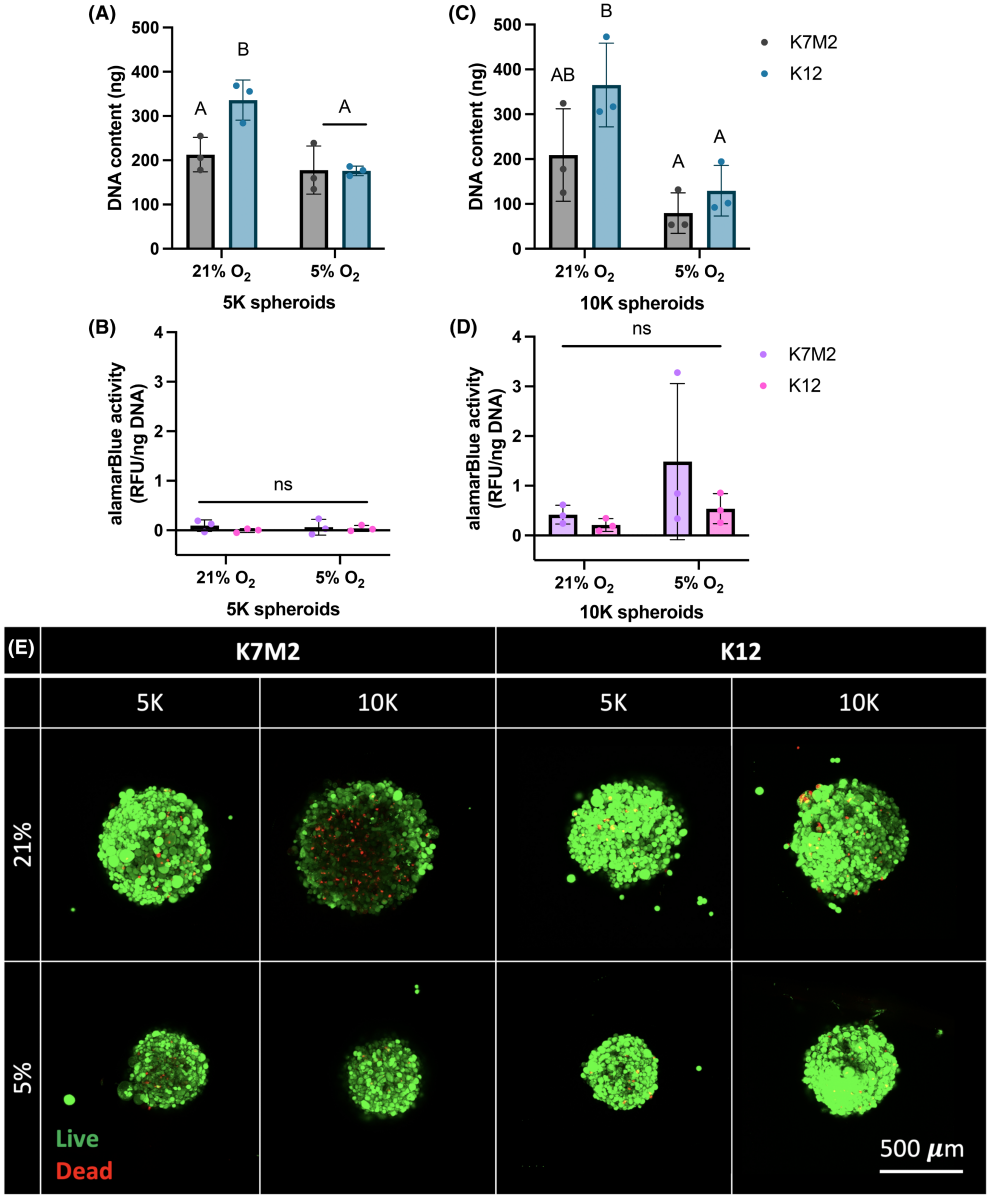
Spheroids formed with 10,000 OS cells have decreased viability. DNA content and metabolic activity of OS spheroids formed with 5,000 (5 K) (A, B) or 10,000 (10 K) (C, D) cells per spheroid. (E) Representative Live/Dead confocal microscopy images of spheroids as max projected z‐stacks after 72 h of formation. Live cells are green, and dead cells are red. Scale bar = 500 μm. Data are mean ± SD (*n* = 3 spheroid wells, 29 spheroids/well). Groups with statistically significant differences based on two‐way ANOVA do not share the same letters; ns denotes no significance among groups.

Due to the observed similarities in spheroid size, we next sought to interrogate spheroid viability 1 day after formation. We evaluated DNA content as an indicator of cell number and overall metabolic activity with an alamarBlue assay. We also directly examined spheroid viability with a Live/Dead assay. After 3 days of formation, DNA content for spheroids formed with 5,000 K12 cells at 21% O_2_ was significantly increased compared to all other 5 K groups (Figure 2A). We noted a similar increase in spheroids formed with 10,000 K12 cells at 21% O_2_ (Figure 2C). This suggests that K12 proliferation is more dependent on oxygen availability compared to K7M2 cells, which were consistently similar in DNA content regardless of cell density and oxygen tension. We also observed that metabolic output for spheroids formed with 5,000 cells is minimal (Figure 2B), whereas for spheroids formed with 10,000 cells, metabolic activity is elevated with an increased trend for those formed under 5% O_2_ compared to 21% O_2_ (Figure 2D). This indicates that spheroids formed with 10,000 cells, especially under hypoxic conditions, upregulate cellular and metabolic function, likely to maintain overall cell survival. This, in turn, suggests that 10,000 cell spheroids may approach the limit of nutrient and oxygen availability, typically considered 100–200 μm in length.^27^, ^28^ These quantitative data are supported by qualitative Live/Dead images, which reveal similar live cell distribution through the spheroid except in spheroids formed with 10,000 K7M2 cells under 21% O_2_ (Figure 2E). Here, we observe pronounced dead cells towards the center of the spheroid, but this is not seen under hypoxic conditions. Together with diameter discrepancies noted between oxygen tensions in Figure 1, this finding implies that during spheroid formation, oxygen sensing and regulation determines optimal spheroid size for nutrient and oxygen diffusion. Perhaps mimicking in vivo tumor formation, the larger K7M2 cells at 21% O_2_ may form then rapidly outgrow their oxygen supply, resulting in the presence of dead cells.

### 3.4 Spheroids of 5,000 OS cells contain more collagen than those of 10,000 OS cells

**FIGURE 3:**
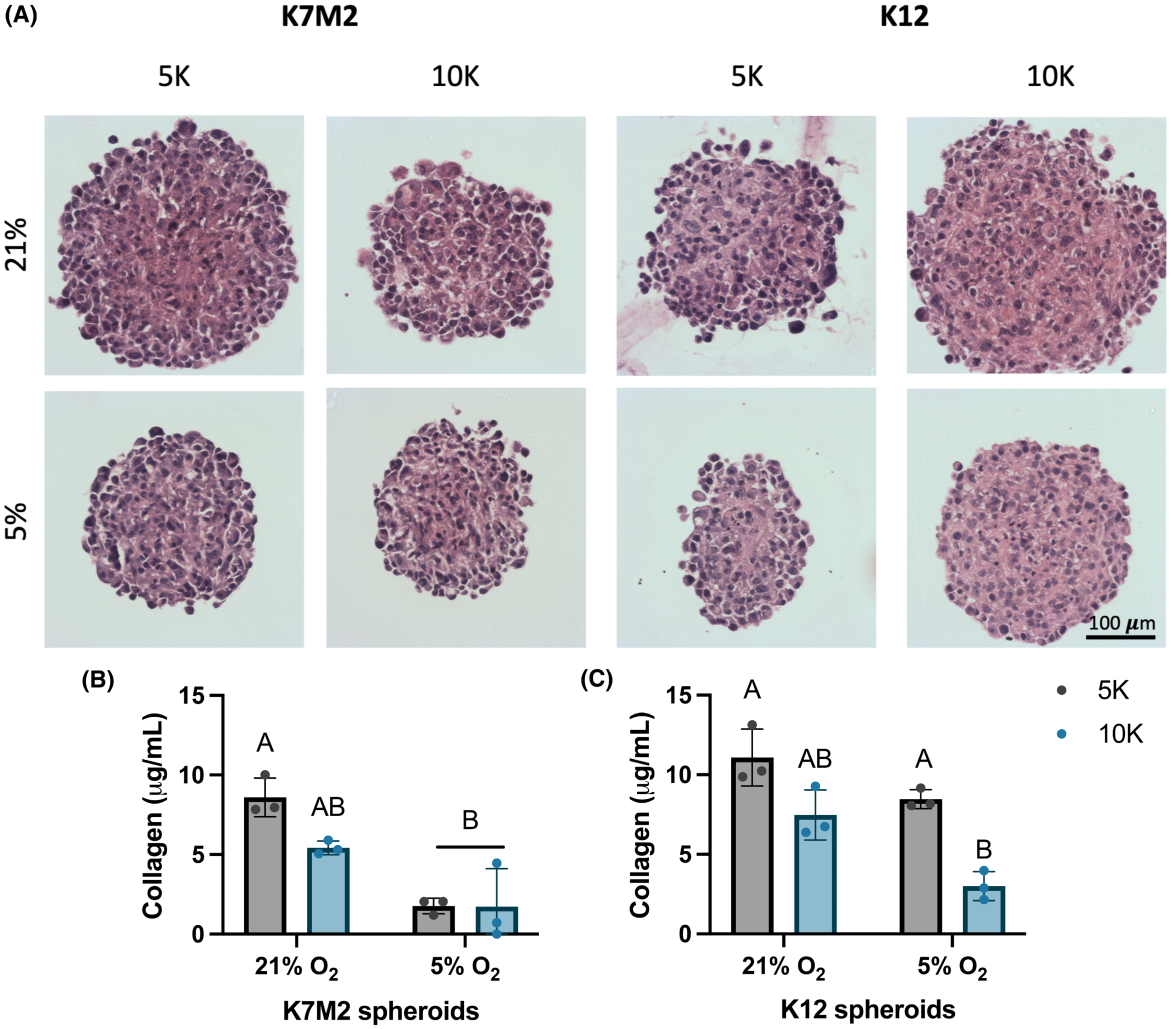
Spheroids formed of 5,000 OS cells contain more collagen than those of 10,000 cells. (A) Representative H&E‐stained spheroids 1 day after formation. Scale bar = 100 μm. Collagen content as measured by hydroxyproline assay for spheroids formed with K7M2 (B) and K12 (C) cells. Data are mean ± SD (*n* = 3 spheroid wells, 29 spheroids/well). Groups with statistically significant differences based on two‐way ANOVA do not share the same letters; ns denotes no significance among groups.

Spheroids are comprised solely of cell mass and endogenous secreted ECM.^26^ Given that spheroids exhibited similar diameters but differences in DNA content, we next aimed to determine if ECM deposition counterbalanced cellular material to yield the observed similarities in spheroid size. To investigate this, we performed H&E staining for general morphology to evaluate cell and matrix distribution. We then directly interrogated ECM content with a hydroxyproline assay to measure collagen, the primary protein component of the ECM.^7^, ^29^ Spheroids stained with H&E exhibited uniform cell and matrix distribution (Figure 3A). Collagen content was often decreased in spheroids formed with 5,000 OS cells (Figure 3B,C), and the trends were roughly inversely proportional to DNA content presented in Figure 2. This confirms that the combined cellular and matrix content within OS spheroids of different cell numbers accounts for similarities in spheroid diameter.

### 3.5 Spheroids of 10,000 K12 cells have increased storage modulus compared to those with 5,000 cells

**FIGURE 4:**
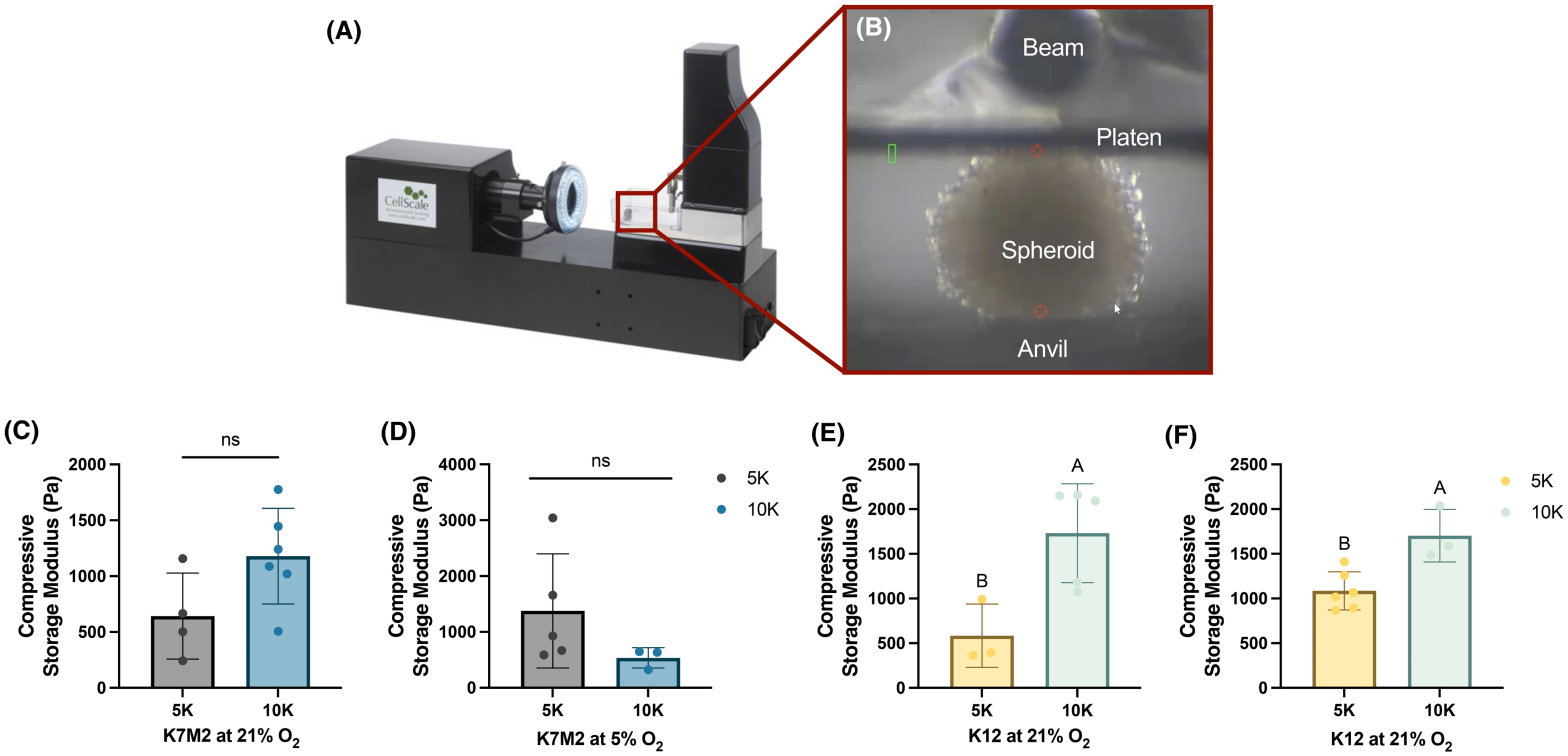
Spheroids of 10,000 K12 cells have increased compressive storage modulus. A MicroTester (A, B) was used to measure the compressive storage modulus of spheroids formed under standard (21% O_2_) (C, E) and physiologic (5% O_2_) (D, F) culture conditions. Data are mean ± SD (*n* = 3–6 single spheroids, distributed over at least three separate wells). Groups with statistically significant differences based on unpaired *t*‐test do not share the same letters; ns denotes no significance among groups.

To further understand how ECM content may affect OS spheroid behavior, we examined individual spheroid compressive storage modulus as a functional output for ECM content. Using a MicroTester (Figure 4A), we loaded single spheroids onto an anvil and compressed them with a platen attached to a beam, which recorded force and displacement (Figure 4B, Video S1). Data were analyzed to yield compressive storage modulus. Compressive modulus exhibited a significant increase in 10 K compared to 5 K spheroids only for K12 spheroids (Figure 4E,F). We did not observe a similar trend in K7M2 spheroids formed under either oxygen tension (Figure 4C,D). Since collagen content in spheroids formed with 10,000 cells was consistently reduced compared to 5 K spheroids, these reported storage moduli were surprising, as collagen is largely thought to provide the structural integrity of most tissues.^29^, ^30^

### 3.6 Spheroids formed with 10,000 K7M2 cells in standard conditions have increased integrin α5 expression

**FIGURE 5:**
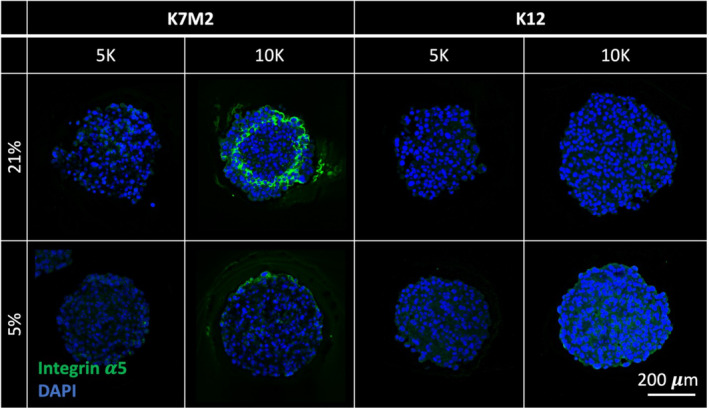
Spheroids formed with 10,000 K7M2 cells cultured under standard conditions have increased integrin α5 expression. Representative confocal microscopy images of sectioned spheroids with immunohistochemical staining for integrin α5 (green) counterstained with DAPI (blue). Scale bar = 200 μm.

To elucidate the direct interactions between OS cells and ECM, we next investigated the role of integrins, which are cell‐ECM binding ligands. Integrins are known to play major roles throughout metastasis, from primary tumor cell egress, attachment to the vascular endothelium, and further migration to metastatic niches.^31^ Capitalizing on the differential metastatic potentials of our cell lines, we chose an integrin subunit that could help delineate a mechanism for these differences and thus OS aggressiveness. Integrin subunit alpha5 (integrin α5) has been heavily investigated as a regulator of stem cell differentiation and migration,^32^ bone formation,^33^ and breast cancer in the context of osseus metastasis.^34^ Though significantly less explored in OS, some studies show that inhibition of integrin α5 with other subunits, such as β1, results in decreased OS migration and proliferation.^35^ Thus, we performed immunohistochemical staining on OS spheroids 1 day after formation for integrin α5 (Figure 5). We found that spheroids formed with 10 K K7M2 cells under standard (21% O_2_) culture conditions had a striking and distinct upregulation of integrin α5 near, but not on, the periphery of the spheroids. Interestingly, this localization corresponds with the Live/Dead staining pattern shown in Figure 2. This again harkens the idea that this particular group of spheroids may mimic in vivo tumor formation, with possible rapid migration away from a hypoxic and nutrient‐depleted core.

We tested spheroids for regions with less than 1.3% O_2_ through pimonidazole treatment and staining to identify if there existed an anoxic region that would prompt the migration pattern we observed. Based on representative confocal microscopy images, we did not observe any areas of positive pimonidazole staining (Figure S3). Thus, none of these spheroids exhibited hypoxic regions with less than 1.3% O_2_, suggesting that an oxygen tension threshold between 1.3%–5% induces OS apoptosis, tumor necrosis, and cell migration away from the region.

### 3.7 Ascorbic acid supplementation does not change OS proliferation or spheroid compressive storage modulus

**FIGURE 6:**
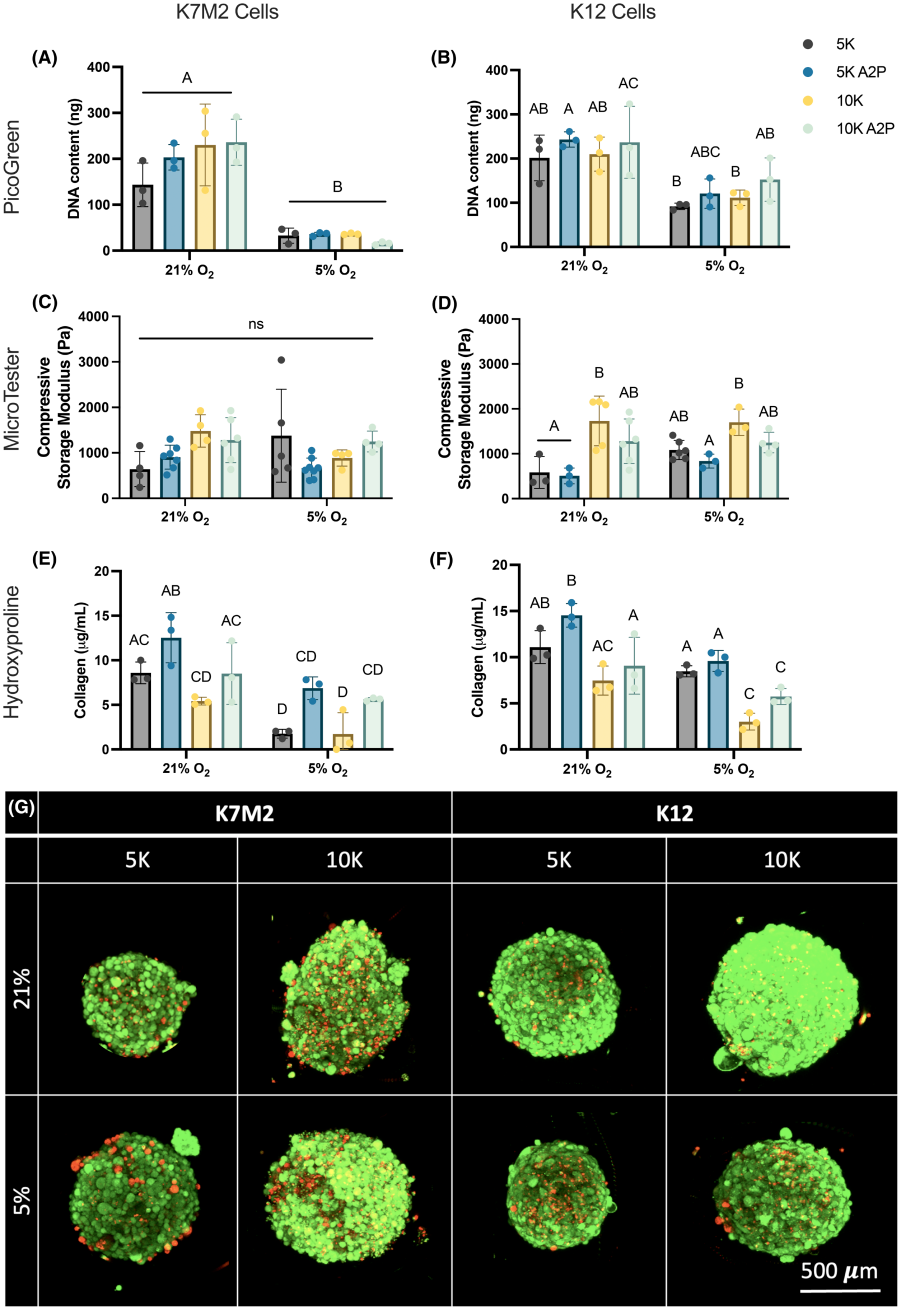
A2P does not change OS proliferation or spheroid compressive storage modulus. Spheroids formed with K7M2 and K12 cells were supplemented with 50 μg/mL A2P during formation to promote ECM deposition. One day after formation, OS spheroid DNA content (A, B), compressive storage modulus (C, D), and collagen content (E, F) were measured. (G) Live/Dead confocal microscopy images, as max projected z‐stacks, of A2P‐supplemented spheroids after 1 day after formation. Live cells are green, dead cells are red. Scale bar = 500 μm. Data are mean ± SD (*n* = 3, spheroid wells, 29 spheroids/well for A, B, E, F; *n* = 3–7, single spheroids, distributed over at least 3 separate wells for C, D). Groups with statistically significant differences based on two‐way ANOVA do not share the same letters; ns denotes no significance among groups.

To interrogate the interplay between matrix deposition and OS behavior, we amplified ECM deposition by supplementing OS spheroids with 50 μg/mL A2P during formation. To characterize OS response, we again evaluated DNA content as an indicator of cell number with PicoGreen (Figure 6A,B), compressive storage modulus as a functional measure of ECM production with the MicroTester (Figure 6C,D), and collagen content with a hydroxyproline assay (Figure 6E,F). We also compared these A2P‐supplemented spheroids with non‐supplemented spheroid data previously shown. Broadly, we detected no statistically significant differences in spheroids formed with A2P across all outputs. However, as validation of the effects A2P supplementation, we did see consistent increasing trends in collagen content in spheroids formed with A2P across all conditions. Interestingly, Live/Dead staining showed increased dead cells across all groups (Figure 6G) compared to non‐A2P supplemented spheroids (Figure 2E), yet live and dead cells are evenly distributed within the spheroid. This perhaps suggests that increased ECM production interferes directly with oxygen and nutrient diffusion or indirectly through interruption of OS cell ability to sense oxygen and nutrient availability. Taken together, these data suggest that ECM production alone does not dictate OS proliferation and bulk tumor mechanical properties but may instead influence molecular signaling processes and subsequent cellular organization.

### 3.8 Increased ECM content increases K7M2 susceptibility to doxorubicin but decreases K12 susceptibility

**FIGURE 7:**
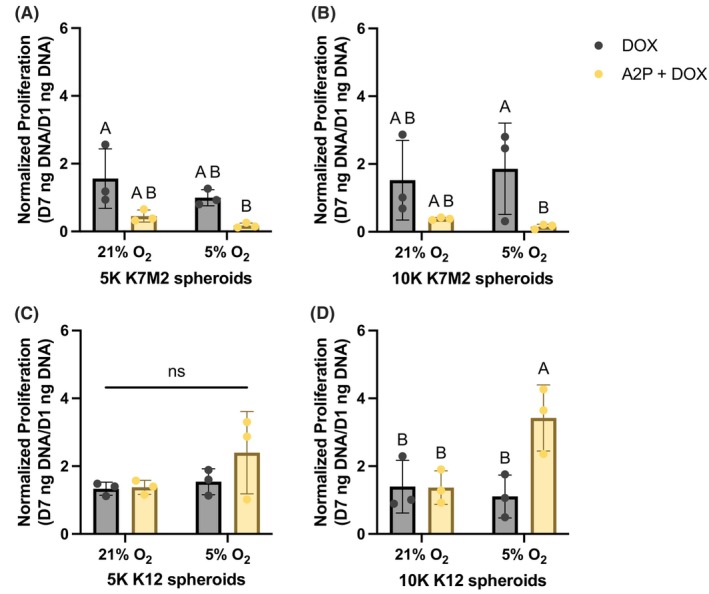
Increased ECM content influences OS spheroid susceptibility to doxorubicin as a function of metastatic potential. OS spheroids without A2P or supplemented with A2P were treated with 0.1 μM DOX for 7 days after formation. DNA content was measured for spheroids formed with K7M2 (A, B) and K12 (C, D) cells. Data are presented as Day 7 DNA content normalized to Day 1 DNA content. Data are mean ± SD (*n* = 3 spheroid wells, 29 spheroids/well). Groups with statistically significant differences based on two‐way ANOVA do not share the same letters; ns denotes no significance among groups.

To enhance the clinical relevance of our findings, we tested OS spheroid response to doxorubicin (DOX). OS spheroids were supplemented with 0 or 50 μg/mL A2P and treated with 0.1 μM DOX for 7 days after formation. DNA content was measured as an indicator of cell number. Data were normalized to DNA content of spheroid groups 1 day after formation to yield normalized proliferation. DOX was not cytotoxic to OS spheroids without A2P, regardless of cell type and culture condition, suggesting that 3D culture as spheroids is inherently chemoprotective. Surprisingly, when spheroids formed with K7M2 cells and A2P were treated with DOX, there were stark decreases in proliferation (Figure 7A,B). However, K12 cells exhibited an entirely different behavior. When supplemented with A2P and treated with DOX, spheroids formed with K12 cells exhibited either the same (Figure 7C) or significantly increased proliferation (Figure 7D). This reveals that K12 cells with excess matrix production have enhanced chemoresistance, especially compared to K7M2s.

## 4 DISCUSSION

It is broadly recognized that cellular processes involved in dedifferentiation, oxygen tension, and ECM deposition and remodeling are major drivers in the development of metastasis.^6^, ^36^ However, little is known about the mechanisms that determine these changes^37^ and if early prognostic indicators specifically related to cellular functions, rather than genetic or familial changes which are historically unreliable for sarcomas,^2^, ^38^, ^39^ may exist. Furthermore, it remains unclear whether aggressive tumors exhibit extensive ECM remodeling or if ECM changes drive tumor aggression. In this work, we studied the interactions between OS cells and ECM in early tumor formation with the use of an OS spheroid model. Specifically, we formed spheroids with highly metastatic murine K7M2 cells and less metastatic K12 cells under standard (21% O_2_) and physiologic (5% O_2_) conditions to delineate differences in early tumor formation and matrix changes as a function of OS cell aggressiveness and oxygen tension.

Our data confirm that spheroid size was largely determined by oxygen tension. Clinically, primary tumor necrosis is a valuable prognostic indicator.^40^ Tumors that have outgrown their vascular and nutrient supply are characterized by low oxygen levels.^1^, ^40^ Indeed, we noted that large K7M2 spheroids formed under 21% O_2_ may show early signs of a necrotic core and cell migration away from that region. Though the clinical literature has no reported oxygen levels within necrotic OS tumors, multiple studies report the upregulation of hypoxia‐associated gene pathways, namely HIF‐1α.^41^, ^42^ Interestingly, HIF‐1α is most often upregulated under short periods of near anoxic levels (<0.1% O_2_),^41^ but our data suggest that aggressive OS tolerance for hypoxia may decrease at levels above 1.3% O_2_. It is possible that the induced migration observed here is due to a well‐documented increase in K7M2 ezrin protein expression,^38^ which has known correlations with hypoxia‐induced cell motility and tumor invasion in other cancers.^43^, ^44^

OS spheroids maximized matrix deposition and cellular content in relation to oxygen and nutrient availability. We evaluated matrix deposition primarily through collagen characterization, yet this oversimplifies in vivo tumor ECM.^36^ Though collagen is the most abundant component of ECM, fibronectin plays an important role in the OS matrix and upregulation is associated with chemoresistance.^45^ Indeed integrin α5 is a known ligand for fibronectin,^32^ and studies with human MG‐63 OS spheroids show increased integrin α5 protein expression with hypoxic treatments.^46^ Furthermore, a hallmark of osteoblastic OS is excessive, immature osteoid deposition with increased hydroxyapatite (HA).^15^ This provides increased substrate stiffness from mineral content and additional cell adhesion and calcium nucleation sites.^47^ Though unexplored here, other studies have shown that HA incorporation in human OS spheroids increases stemness markers and HIF‐1α.^12^ Unfortunately, these cell lines are not osteogenic despite their osteoblastic origins, representing a limitation of this work.

The combined effects of hypoxia and matrix deposition reported here are also important to consider. As a sarcoma, clinical ECM deposition is so abundant that it effectively blockades the tumor and inhibits immune cell infiltration.^6^, ^7^ This process creates pockets of lower, but not anoxic, oxygen tension within tumors, where numerous hypoxia‐related pathways in addition to HIF‐1α become upregulated.^12^ Multiple studies report that associated changes drive a more aggressive, though less differentiated phenotype.^48^ Within cancer, tumor cell dedifferentiation is largely referred to as the epithelial to mesenchymal transition, where an upregulation in stem cell‐associated genes, such as NANOG, OCT‐4, and SOX‐2, induce a phenotype that is broadly known as a cancer stem cell (CSC).^6^, ^48^ CSCs are known to be more chemoresistant, yet often enter a quiescent state in hypoxic environments.^6^, ^48^ Specifically in OS, studies show that NOTCH signaling and associated increases in aldehyde dehydrogenase (ALDH) expression are reliable markers of stemness.^49^ K7M2s, under standard culture conditions, have increased NOTCH signaling, yet hypoxia is known to upregulate NOTCH ligand expression.^49^ As K12s show increased differentiation compared to K7M2s under baseline conditions,^38^ it is possible that 5% O_2_ culture conditions may effectively increase K12 stemness. This would correspond with our observations that K12 spheroids supplemented with A2P at 5% O_2_ appear more chemoresistant, even compared to K7M2s under the same conditions due to relative changes in NOTCH expression. However, another possible mechanism to explain this finding is that ECM composition may also differ between cell lines, where K7M2s may produce more fibronectin. As discussed already, this is indirectly supported by the integrin α5 staining. These differences may promote subtle changes in diffusion that could impact chemotherapeutic resistance, and thus warrants further investigation with mass spectrometry. As such, this model could serve as an effective platform to investigate CSC‐related changes as a function of oxygen tension and differentiation.

In conjunction with future studies that further evaluate ECM changes, OS‐mediated matrix degradation and remodeling, and associated genetic changes, our findings provide a foundation for OS ECM research on prognostic indicators. However, there are notable limitations beyond those already mentioned. As cell lines, K7M2 and K12 cells have been selected for culture under 21% O_2_ during isolation.^50^ This renders hypoxic studies less accurate. Additionally, the use of homotypic spheroids fails to capture important crosstalk between cell types. Future directions for this work may include co‐culture with fibroblasts to better understand the interplay between OS signaling and fibroblast‐driven changes to ECM production.

Overall, this study shows that ECM production and oxygen tension regulate OS behavior. Specifically, these two factors are influential determinants of spheroid size through cell organization based on nutrient and oxygen distribution, and we also show that these responses are generally not affected by metastatic potential. The exceptions to this are (1) spheroids of 10,000 K7M2 cells formed in 21% O_2_ feature unique viability localizations and cell migration patterns and (2) spheroids formed with 10,000 K12 cells and A2P under 5% O_2_, which, when treated with DOX, show increased chemoresistance, may be due to a hypoxia‐induced increase in stemness. These studies are then contextualized as they relate to key clinical markers, such as HIF‐1α, tumor ECM and mineral content, cancer stem cells, and chemo‐responsiveness. This work presents OS spheroids as an excellent platform for further research to mechanistically study ECM‐OS interactions. Further, after extensive validation, spheroids formed with primary tumor isolates have the potential to provide much needed, accurate personalized drug screening, as well as other mechanistic investigations for the discovery of novel clinical therapeutic targets and prognostic indicators.

## AUTHOR CONTRIBUTIONS

**Isabel S. Sagheb:** Conceptualization (supporting); data curation (equal); investigation (equal); writing – review and editing (supporting). **Thomas P. Coonan:** Conceptualization (supporting); data curation (equal); investigation (equal); methodology (supporting). **R. Lor Randall:** Conceptualization (supporting); funding acquisition (lead); writing – review and editing (equal). **Katherine H. Griffin:** Conceptualization (equal); data curation (lead); formal analysis (equal); investigation (equal); methodology (equal); writing – original draft (lead); writing – review and editing (equal). **J. Kent Leach:** Conceptualization (equal); funding acquisition (lead); investigation (supporting); methodology (supporting); project administration (lead); resources (lead); supervision (lead); writing – review and editing (lead).

## CONFLICT OF INTEREST STATEMENT

The authors declare no conflicts of interest.

## Supporting information


**Video S1.** Representative video of mechanical testing of an osteosarcoma spheroid (K12 spheroid formed of 10,000 cells at 21% O_2_) on the MicroTester.

Data S1.

## Data Availability

The data that support the findings of this study are available from the corresponding author upon reasonable request.
