# Supplementary material for: Extracellular matrix production and oxygen diffusion regulate chemotherapeutic response in osteosarcoma spheroids
Source: Cancer Med. 2024 Sep 20;13(18):e70239. doi: 10.1002/cam4.70239 (PMC11413413; doi:10.1002/cam4.70239)
Supplement: Supplementary file 2 — Data S1. [file CAM4-13-e70239-s001.docx]

SUPPLEMENTARY INFORMATION FOR

Title: Extracellular matrix production and oxygen diffusion regulate chemotherapeutic response in osteosarcoma spheroids

Authors: Isabel S. Sagheb^1ᴓ^, Thomas P. Coonan^1ᴓ^, R. Lor Randall^2^, Katherine H. Griffin^2,3^, J. Kent Leach^1,2^

Affiliations

^1^Department of Biomedical Engineering, University of California, Davis, California, USA

^2^Department of Orthopaedic Surgery, UC Davis Health, Sacramento, California, USA

^3^School of Veterinary Medicine, University of California, Davis, California, USA

^ᴓ^Authors contributed equally


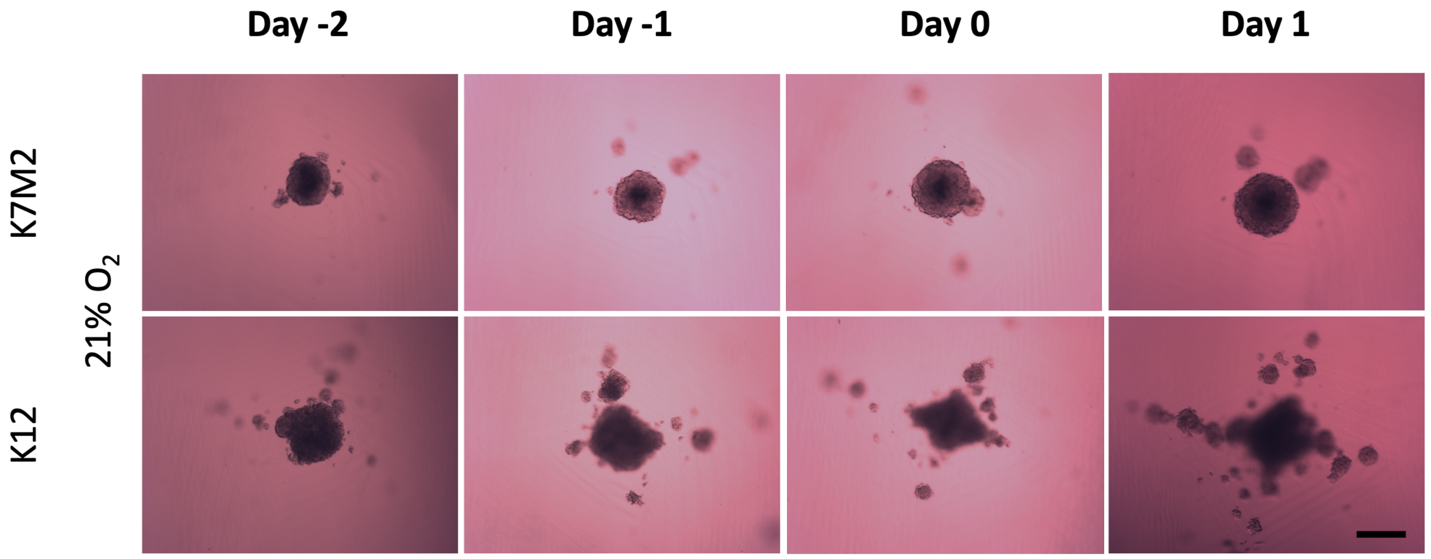


**Figure S1:** Representative images of OS spheroids formed with 5,000 cells in Aggrestamp microwells. Scale bar = 500 μm.

**Figure S2:** Spheroid diameters compared across oxygen tension, where every point is the average diameter for one cell type at each day of formation. Data are mean ± SD (n=16; 2 cell types, 4 days of formation). Groups with statistically significant differences based on non-paired t-test do not share the same letters. p=0.0069.


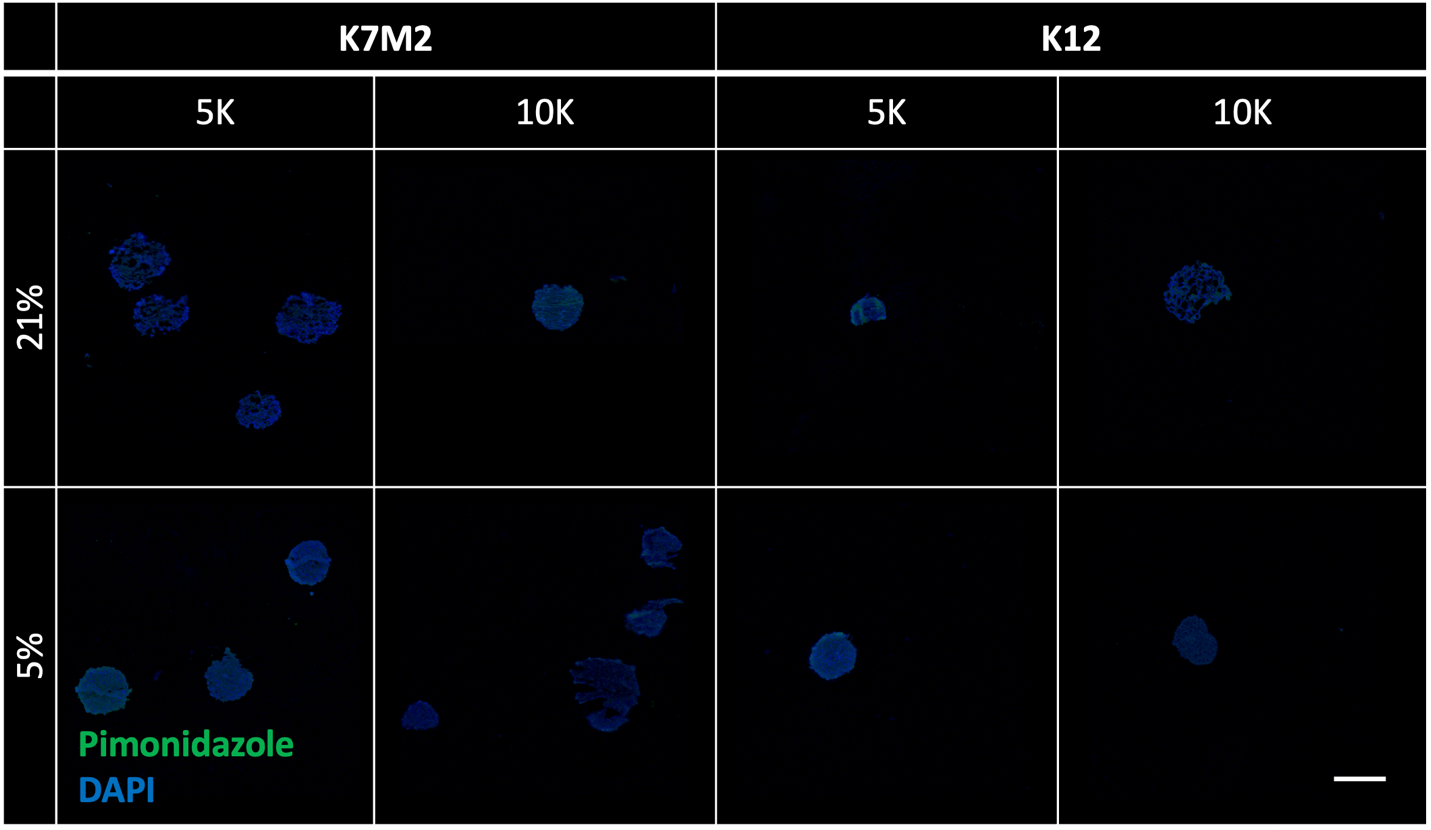


**Figure S3.** Representative confocal microscopy images of fixed and sectioned OS spheroids stained with anti-pimonidazole (green) to identify areas with less than 1.3% O_2_ and counterstained with DAPI (blue). Scale bar = 200 μm.
